# Mindfulness and Voluntary Work Behavior: Further Support for an Affect Mediation Model

**DOI:** 10.3389/fpsyg.2022.742221

**Published:** 2022-05-12

**Authors:** Michael D. Robinson, Sukumarakurup Krishnakumar

**Affiliations:** ^1^Department of Psychology, North Dakota State University, Fargo, ND, United States; ^2^Department of Management, Keck Graduate Institute, Claremont, CA, United States

**Keywords:** trait mindfulness, job affect, job engagement, work deviance, citizenship behavior

## Abstract

Mindfulness, defined in terms of greater attention and awareness concerning present experience, seems to have a number of psychological benefits, but very little of this research has focused on possible benefits within the workplace. Even so, mindfulness appears to buffer against stress and negative affect, which often predispose employees to deviant behaviors. Conversely, mindful employees may be more engaged with their jobs, which could support organizational citizenship. Two studies (total *N* = 418) pursued these ideas. In Study 1, part-time employees who were higher in dispositional mindfulness were less prone to job negative affect, which in turn predicted lower levels of workplace deviance. In Study 2, more mindful full-time employees were more engaged, and less stressed, and these variables mediated a portion of the relationship between mindfulness and organizational citizenship. Collectively, the two studies link mindfulness to both traditional forms of voluntary work behavior while highlighting mediational pathways.

## Introduction

Mindfulness, which can be defined in terms of receptivity to current moments of experience, can be trained through the use of meditation-related interventions (Creswell, 2017) or it can vary in naturalistic terms, such that some individuals, relative to others, more routinely interact with the environment in ways that are conscious and aware (Brown and Ryan, 2004). Whether manipulated or naturally varying, the correlates of mindfulness appear to be similar and they are traditionally thought of in terms of reduced levels of stress and distress in combination with higher levels of psychological wellbeing (Goodman et al., 2015). Increasingly so, however, researchers have explored the interface between mindfulness and social behavior, with results appearing to suggest that mindful individuals are less prone to antisocial behavior (DeSteno et al., 2018) and more prone to prosocial behavior (Schindler and Friese, 2022). The scope of these behavioral correlates is a topic of considerable interest (Condon, 2019) and understanding these relationships may require increased attention concerning plausible mediators or mechanisms (Reb et al., 2020; Schindler and Friese, 2022). The present research sought to contribute to this understanding in the particular context of voluntary work behaviors, which include tendencies toward workplace deviance (Bennett and Robinson, 2003) as well as organizational citizenship (Organ, 2018). We will suggest that the affective correlates of mindfulness (Goodman et al., 2015) may play a significant role in linking mindfulness to these workplace outcomes.

## Literature Review

Mindfulness can counteract a number of processes that generate negative affect, such as worry or rumination (Teasdale and Chaskalson, 2011), which cannot be sustained when the person maintains a focus on present-moment experience (Brown and Ryan, 2004). In addition, mindfulness can stimulate interest in what one is doing (Lyddy and Good, 2017), which can give rise to forms of positive affect that benefit from engagement (Ryan et al., 2021). Mindfulness also allows for more attuned forms of emotion regulation (Teper et al., 2013), which can be used to regulate both positive and negative affective states (Roemer et al., 2015). Hence, many of the benefits of mindfulness should be experienced in subjective (or affect-related) terms (Brown et al., 2007; Goodman et al., 2015). Consistent with this analysis, a recent meta-analysis has linked variations in dispositional mindfulness to higher levels of confidence and life satisfaction and to lower levels of stress, negative emotion, anxiety, and depression (Mesmer-Magnus et al., 2017). Relatedly, there is a robust relationship between mindfulness and self-esteem and this relationship has been shown to mediate some of the variance linking mindfulness to subjective states of happiness (Bajaj et al., 2019).

What mindfulness does to behavior is less certain at the present time, but this is an emerging area of interest (Ryan et al., 2021; Schindler and Friese, 2022). Perhaps most straightforwardly, mindfulness should promote better awareness of what one is doing (Lyddy and Good, 2017), which should decrease tendencies toward behavioral error in tasks dependent on attentional resources (Kang et al., 2013). Being in a mindful state should also render it more likely that one is the voluntary author of one’s behaviors, relative to acting on “autopilot” or reacting in an invariant stimulus–response manner (Kang et al., 2013). In this respect, Ryan et al. (2021) contend that mindfulness supports autonomous motivation, meaning that the individual is more likely to fully endorse their behaviors and/or their behaviors are more likely to follow from core values of the self, relative to external pressures. This perspective has been supported by data linking mindfulness to the endorsement of self-aligned reasons for one’s behavior (Donald et al., 2020), though the exact behaviors that would follow from higher levels of autonomous motivation are not entirely clear.

An analysis of antisocial (e.g., aggressive) behavior suggests that there are two forms of it—a proactive form and a reactive form (Hubbard et al., 2010). Some individuals engage in proactive aggression as a way of forcing others to do things that benefit the self (Crick and Dodge, 1996). This form of aggression is not extremely common (Raine et al., 2006) and it is psychopathic in nature (Furnham et al., 2013). The reactive form of aggression is more common (Raine et al., 2006) and it is exhibited by many individuals under certain circumstances—namely, those that involve high levels of anger or negative affect (Berkowitz, 1983). Because mindful people are less prone to anger and negative affect (Brown and Ryan, 2003), and also because higher levels of mindfulness support higher levels of self-control (Fetterman et al., 2010), one would expect mindful individuals to be less prone to reactive aggression and there is evidence consistent with this point. For example, Heppner et al. (2008) found that mindfulness was inversely correlated with trait aggression and mindfulness-based interventions have also proven themselves to be useful in mitigating violent tendencies within clinical populations (Gillions et al., 2019). In Study 1 of the present paper, we will examine whether mindfulness is similarly protective against deviant workplace behaviors, which often have a reactive signature to them (Bennett and Robinson, 2003).

As indicated above, a number of studies have linked mindfulness to prosocial behavior (Donald et al., 2019), though the scope of these effects as well as their mechanisms require further study (Reb et al., 2020; Schindler and Friese, 2022). Mindfulness is protective against stress and distress (Mesmer-Magnus et al., 2017), which often motivate individuals to escape situations that involve others in need (Batson, 2011). Mindfulness may also give rise to thoughts and feelings that often precipitate helping behavior, such as empathetic concern (Batson, 2011). Consistent with this analyses, Berry et al. (2018) reported four studies in which mindful individuals were more prosocial toward ostracized strangers and Hafenbrack et al. (2020) showed that a mindfulness-based intervention gave rise to higher levels of empathy and perspective taking, consistent with the long-standing contention that the mindful mode of being is a compassionate one (Davidson and Harrington, 2002). In this connection, we can also highlight data suggesting that mindful individuals are more engaged with their environments (Gunasekara and Zheng, 2019), which should encourage proactivity in helping individuals who would benefit from this help (Rich et al., 2010). In Study 2 of the present paper, we will examine whether mindfulness supports prosocial behavior within the workplace, as captured by the organizational citizenship construct (Organ, 2018).

Having reviewed lines of research pertinent to the present project, we note that questions of mechanism are critical in mindfulness research (Reb et al., 2020; Schindler and Friese, 2022), and there is a large body of work linking mindfulness to affective variables such as stress, anxiety, anger, confidence, and so on (Mesmer-Magnus et al., 2017; Carpenter et al., 2019). Given that this is true, there is also a growing sense that mindfulness may impact behaviors largely or primarily through affective routes. This sort of *affect mediation model* has been most extensively applied within the addictive behavior realm (for a review, see Heppner et al., 2015). In such research, mindfulness has been shown to be beneficial in curtailing problematic habits such as alcoholism and cigarette smoking because it protects against the negative feelings—such as stress, craving, and depression—that often precipitate these behaviors within addicted populations (Heppner et al., 2015). As an example, Black et al. (2012) found that individuals with higher levels of trait mindfulness were less likely to smoke cigarettes in part because they were less prone to stress, anger, and depressive affect. Because similar results have been reported in the context of other addictive behaviors (e.g., Witkiewitz and Bowen, 2010). Heppner et al. (2015) proposed that mindfulness protects against addictive behaviors *because* it protects against the negative feelings that often trigger these behaviors.

Krishnakumar and Robinson (2015) proposed that similar processes could explain some of the benefits of mindfulness within the workplace. However, there a need for more empirical research on relations between mindfulness and organizational outcomes (Reb et al., 2020) and this may be particularly true with respect to variations in dispositional mindfulness, relative to mindfulness-based interventions (Kersemaekers et al., 2018). Furthermore, there is a lack of research on contextual workplace behaviors (Organ, 2018), relative to other outcomes such as job performance (Dane, 2011). With respect to contextual workplace behaviors, it may be that mindful employees are better employees because they are less prone to the feelings that give rise to hostile workplace behaviors and/or because they are more prone to the feelings that give rise to prosocial workplace behaviors. In this context, though, it is important to note that there have not been *any* full applications of the affect mediation model of dispositional mindfulness to contextual workplace behaviors, which represents a gap in the literature.

Accordingly, the research problem that was investigated involved a full application of the affect mediation model to contextual workplace behaviors, which are voluntary workplace behaviors that may be key to the workplace but are not requirements of most jobs (Borman and Motowidlo, 1997). To fully apply the model, we assessed variations in dispositional mindfulness in the context of both deviant workplace behaviors (Study 1) and organizational citizenship behaviors (Study 2). We sought to determine whether mindfulness relates to each class of behaviors and we also sought to investigate whether affect-related variables play a role in mediating such relationships. Study 1 measured positive and negative affect within the workplace, whereas Study 2 examined the broader affective categories of job stress and job engagement. The latter experiences, in particular, might play a role in mediating relationships between mindfulness and contextual workplace behavior (Mesmer-Magnus et al., 2017).

## Hypotheses Development

Deviant workplace behaviors—such as getting into fights with coworkers or stealing from the workplace—are often precipitated by negative feelings within the workplace (Spector, 2011). Mindfulness seems to be protective against negative feelings, both generally (Brown et al., 2007) and also within the workplace (Mesmer-Magnus et al., 2017; Lomas et al., 2019). Because this is true, mindful employees should be less prone to deviant workplace behaviors. Furthermore, and according to an affect mediation model, this inverse relationship between dispositional mindfulness and workplace deviance should be mediated by lower levels of job negative affect. In other words, negative affect should explain some of the variance linking mindfulness to lesser deviance (MacKinnon and Fairchild, 2009). Study 1 was conducted to test these predictions.

*Hypothesis 1*: Mindfulness will inversely predict workplace deviance (Study 1).

*Hypothesis 2*: Job negative affect will mediate mindfulness/deviance relationships (Study 1).

While Study 1 focused on deviant workplace behaviors, Study 2 focused on organizational citizenship behaviors (OCBs). Whereas deviant workplace behaviors tend to harm an organization or its members (Bennett and Robinson, 2003), OCBs tend to help an organization and its members (Organ, 2018). Pertinent to this second focus, there are reasons for thinking that mindful employees may engage in OCBs more often. In particular, mindfulness has been shown to be protective against job stress (e.g., Lomas et al., 2019) and job stress tends to undermine organizational citizenship (Greenidge and Coyne, 2014). In addition, mindful employees should be more engaged (attentive and interested) in the conduct of their jobs (Malinowski and Lim, 2015) and workplace engagement has been shown to give rise to higher OCB frequencies (Rich et al., 2010). Thus, the feelings linked to stress and engagement should play some role in mediating the relationship between mindfulness and tendencies toward organizational citizenship. Study 2 was designed to test these predictions.

*Hypothesis 3*: Mindfulness will positively predict organizational citizenship (Study 2).

*Hypothesis 4*: Job stress and engagement will mediate relationships between mindfulness and citizenship behavior (Study 2).

In summary, we conducted two studies to examine an affect mediation model that may be capable of explaining links between dispositional mindfulness and voluntary workplace behaviors. The first study focused on the role of negative feelings in mediating relationships between mindfulness and deviant workplace behaviors and the second study focused on the roles of job stress and engagement in mediating relationships between mindfulness and organizational citizenship behaviors. Together, the two studies sought to be comprehensive in a manner that one study could not be. Other similarities and differences among the studies will be noted during the course of their presentation.

## Study 1: Mindfulness, Negative Affect, and Workplace Deviance

### Method

#### Participants and Procedures

To gain initial insights into the processes of interest, we focused on undergraduate student employees, both because this sample was convenient and because many undergraduates have fairly substantial jobs. These participants, who received course credit at a medium-sized Midwestern university, had to be working at least 20 h per week to qualify for the study (or else they were excluded, though there were no other exclusion criteria) and we managed to recruit 89 of them (53% female; 86% Caucasian; *M* age = 21.14) during the course of a semester. The student employees had been working at their current jobs for an average of 15.23 months (range: 1–108 months) and were employed in diverse fields such as accounting, customer service, healthcare, and manufacturing. The average level of job autonomy was comparable to some full-time jobs (*M* = 3.57; *SD* = 0.90; *α* = 0.85), as captured by a factual autonomy scale (Spector and Fox, 2003) included for descriptive purposes.

The study itself was completed online through a Qualtrics-programmed website. Participants were directed to this website through the university’s participant pool platform and confidentiality was maintained through the use of de-identified codes. The order of the measures was randomized and different sets of participants received different randomized orders.

#### Measures

##### Dispositional Mindfulness

The Mindful Attention-Awareness Scale (MAAS: Brown and Ryan, 2003) is the most frequently used dispositional mindfulness scale (Medvedev et al., 2016) and we sought to use this well-validated scale (Quaglia et al., 2015; Bajaj et al., 2016) as a basis for the present research, in part to contribute to cumulative progress in the field (Mesmer-Magnus et al., 2017). The MAAS consists of 15 items (e.g., “I break or spill things because of carelessness, not paying attention, or thinking of something else” & “I do jobs or tasks automatically without being aware of what I’m doing,” both reverse-scored) that are rated with respect to how frequently they occur to the self (1 = almost never; 6 = almost always) in a single-factor way that is thought to capture the theoretical core of mindfulness (Brown and Ryan, 2004). Empirically, the MAAS has been shown to predict states of mindfulness in daily life (Brown and Ryan, 2003) as well as a number of theory-consistent physiological and psychological outcomes (Brown et al., 2007; Goodman et al., 2015). A total score was computed by averaging across items (*M* = 3.95; *SD* = 0.99; *α* = 0.94).

##### Job Negative (and Positive) Affect

People who experience job negative affect (JNA) are more likely to “act out” in deviant or counterproductive ways (Fox et al., 2001; Spector, 2011). To capture such feelings, we administered the Job-related Affective Well-being Scale (JAWS), which was specifically designed to measure affective states at the workplace (Van Katwyk et al., 2000). The scale accomplishes this by pairing 15 negative (e.g., angry and frustrated) and 15 positive (e.g., cheerful and elated) markers with the phrase “My job made me feel…” (e.g., “my job made me feel frustrated”). Participants indicated the frequency with which (1 = never; 5 = extremely often) they experienced each feeling on the job in the last 30 days. We calculated separate scores for JNA (*M* = 2.43; *SD* = 0.74; *α* = 0.91) and JPA (*M* = 3.08; *SD* = 0.79; *α* = 0.95).

##### Deviant Work Behaviors

Deviant work behaviors are problematic, non-normative behaviors that can create dysfunction within the workplace when they are too prevalent (Bennett and Robinson, 2003). There are two primary forms of deviance termed interpersonal, which targets other individuals, and organizational, where the target seems to be the organization as a whole (Mackey et al., 2021). These behaviors are often assessed by self-report not only for the sake of convenience, but also because employees are often in a unique position to report on some of these behaviors (e.g., drug use or theft: Spector and Fox, 2002). Indeed, on the basis of their meta-analytic results, Berry et al. (2012) concluded that self-reports of deviance seem to have greater validity than other-reports. In Study 1, deviance was assessed using the Bennett and Robinson (2000) measure, which asked employees to report on the frequency with which (1 = never; 7 = daily) they had engaged in specific sorts of behaviors over the previous year. Seven of the items assessed interpersonal deviance (e.g., “cursed at someone at work”; *M* = 1.78; *SD* = 1.09; *α* = 0.92) and 12 assessed organizational deviance (e.g., “used an illegal drug or consumed alcohol on the job”; *M* = = 1.73; *SD* = 0.96; *α* = 0.94).

#### Analysis Software

Initial analyses were performed with SAS 9.4 software. Mediation analyses were performed with a PROCESS-based macro (Hayes, 2013) that was created for use with SAS software. This software platform was used for both studies.

### Results

#### Correlations Among Variables

Mindful employees were less likely to engage in interpersonal deviance (e.g., by insulting coworkers), *r* = −0.31, *p* = 0.004, and they were less likely to engage in organizational deviance (e.g., by stealing items from work), *r* = −0.34, *p* = 0.001. Affective states were also consequential in that higher levels of negative affect were associated with higher levels of interpersonal deviance, *r* = 0.53, *p* < 0.001, as well as higher levels of organizational deviance, *r* = 0.43, *p* < 0.001. Positive affect on the job, by contrast, did not predict interpersonal deviance, *r* = −0.12, *p* = 0.283, or organizational deviance, *r* = −0.05, *p* = 0.621. Finally, there were reasons for thinking that mindfulness could mitigate deviance through an affective route, as hypothesized. Specifically, there was a fairly strong inverse relationship between mindfulness and job negative affect, *r* = −0.47, *p* < 0.001, though mindfulness was not a significant predictor of job positive affect, *r* = 0.11, *p* = 0.325.

#### Mediational Results Involving Interpersonal Deviance

Mindful employees might be less prone to interpersonal deviance *because* they are less prone to feelings such as frustration or anger on the job. To formally examine such ideas, we used the PROCESS-related procedures of Hayes (2013), which employ bootstrapping methods (MacKinnon and Fairchild, 2009) to determine the significance of mediational pathways. For the sake of comprehensiveness, we retained both JPA and JNA in these models, though the conclusions would be substantively the same in JNA-only models. All variables were standardized to aid magnitude interpretation.

As shown in the top portion of Table 1, JNA emerged as a plausible mediator of the relationship between mindfulness and interpersonal deviance. Mindfulness was inversely related to JNA, the proposed mediator (Model 1), and it was inversely related to interpersonal deviance, the outcome to be explained (Model 4). Further, in the model including both mindfulness and the affective variables as predictors (Model 3), JNA remained a significant predictor of interpersonal deviance, and mindfulness was no longer a significant predictor. These results suggest that mindful employees are less prone to interpersonal deviance because they are less prone to the negative emotional feelings that often precipitate such behaviors (Bennett and Robinson, 2003).

**Table 1:** PROCESS model results for interpersonal deviance (top panel) and organizational deviance (bottom panel), Study 1.

		Outcome	Predictor	*β*	*t*	*p*
*Predictor to Mediator Models*						
	Model 1	JNA	Mind.	−0.47	−4.99	<0.01
	Model 2	JPA	Mind.	0.11	0.99	0.32
*Full Mediational Model*						
	Model 3	IP Dev.	Mind.	−0.07	−0.64	0.52
			JNA	0.50	4.78	<0.01
			JPA	−0.05	−0.59	0.56
	Model 4	IP Dev.	Mind.	−0.31	−3.00	<0.01
*Full Mediation Model*						
	Model 3	Org. Dev.	Mind.	−0.17	−1.65	0.10
			JNA	0.33	3.10	<0.01
			JPA	0.00	0.03	0.98
*Predictor to Outcome Model*						
	Model 4	Org. Dev.	Mind.	−0.33	−3.38	<0.01

Mind, Mindfulness; JNA, Job Negative Affect; JPA, Job Positive Affect; IP Dev., Interpersonal Deviance; and Org. Dev., Organizational Deviance.

**Figure 1:**
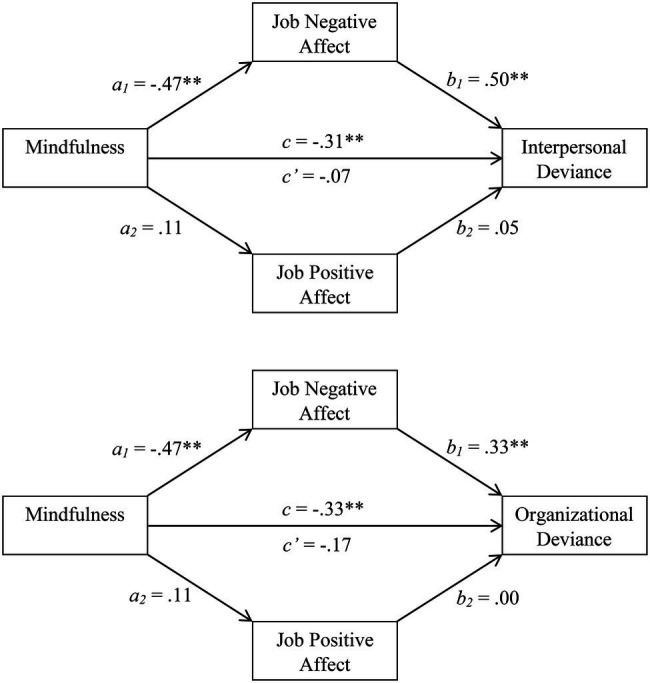
Job affect as a mediator of the relationship between mindfulness and interpersonal deviance (**top panel**) and organizational deviance (**bottom panel**), Study 1.

The results can also be understood in graphic terms. As shown in the top panel of Figure 1, mindfulness predicted JNA (path *a_1_*) and JNA predicted interpersonal deviance (path *b_1_*). When accounting for such pathways, the magnitude of the relationship between mindfulness and interpersonal deviance dropped from −0.31 (path *c*) to −0.07 (path *c’*). To determine whether this suggestion of mediation was significant, we turned to the bootstrapping analyses (Hayes, 2013). The mean estimate for the combined *ab* pathways was −0.24 and the 95% bias-corrected confidence interval (BCCI) for these combined pathways excluded 0 (−0.43 to −0.11). Thus, there was significant evidence for affective mediation. Further analyses, however, revealed that this mediation was primarily due to JNA (*a_1_b_1_ M* = −0.23; 95% BCCI = −0.42 to −0.11) relative to JPA (*a_2_b_2_ M* = −0.01; 95% BCCI = −0.06 to 0.01).

As another way of understanding the mediation-related results, we compared the magnitude of the *ab* (indirect) and *c* (total) pathways (Hayes, 2013). For the model as a whole (Figure 1), 78.31% of the total effect of mindfulness on interpersonal deviance could be ascribed to emotional experiences within the workplace. This is a fairly substantial percentage of variance (MacKinnon and Fairchild, 2009; Hayes, 2013). Further analyses, though, indicated that the indirect pathway involving JNA was responsible the bulk of this variance (76.43%), relative to the indirect pathway involving JPA (1.88%).

#### Mediational Results Involving Organizational Deviance

We next sought to determine whether similar processes are operative in the context of organizational deviance. As indicated by Model 4 within the bottom section of Table 1, mindfulness was a negative predictor of organizational deviance. At least a portion of this relationship could be due to affective states, particularly given that mindfulness was protective against negative affect (Model 1, which is identical for both regression sets). Further evidence for this possibility arises when we compare Models 3 and 4 (also see the bottom panel of Figure 1). The direct effect of mindfulness on organizational deviance (*c’* = −0.17) was smaller than the total effect (*c* = −0.33), and negative affect predicted organizational deviance with mindfulness controlled (*b_1_* = 0.33). It therefore appears that JNA, though not JPA (*b_2_* = 0.00), might mediate the relationship between mindfulness and organizational deviance.

Bootstrapping results provided convergent support. The mean estimate for the combined indirect effect (*ab*) was −0.15 and the 95% BCCI was −0.31 to −0.05. Because this interval excludes 0, we can conclude that the mediational model was significant (MacKinnon and Fairchild, 2009). Further analyses, though, revealed that the *a_1_b_1_* pathway was significant (estimated *M* = −0.16; 95% BCCI = −0.31 to −0.05), whereas the *a_2_b_2_* pathway was not (estimated *M* = 0.00; 95% BCCI = −0.03 to 0.03). Thus, in keeping with similar results involving interpersonal deviance, JNA, relative to JPA, was the more consequential mediator. Our final computations revealed that 47.08% of the total effect of mindfulness on organizational deviance could be ascribed to affective factors (46.99% involving the JNA route and 0.08% involving the JPA route). Overall, then, Study 1 converges on at least two conclusions: Mindful people are less prone to acts of workplace deviance, and this is largely so because they are less prone to JNA.

### Discussion

Beyond establishing a role for mindfulness at work, we also sought to contribute to an understanding of the mechanisms involved. Consistent with an emotion-mediation framework (Heppner et al., 2015; Krishnakumar and Robinson, 2015), Study 1 found that mindful employees were quite a bit less prone to negative emotional experiences on the job, which should spare them from job-related outcomes that are precipitated by negative affect (Spector, 2011). In concert with this idea, job negative affect was found to mediate the mindfulness/deviance relationship such that it became weaker, and in fact non-significant, when controlling for job affect. These results point to affective experiences as a key reason for expecting mindfulness to operate as it does. We sought to extend this analysis in Study 2.

## Study 2: Mindfulness, Stress, Engagement, and Citizenship Behavior

Study 2 sought to extend the present affect mediation perspective of mindfulness to another prominent class of voluntary workplace behaviors—namely, organizational citizenship behaviors (OCBs). Like deviant workplace behaviors, organizational citizenship behaviors are often motivated by affective experiences or feeling states (Shockley et al., 2012), some of which motivate such behaviors (e.g., engagement) and some of which do not (e.g., stress). According to an affect mediation perspective, mindful individuals should be more prone to the feelings that support OCBs, which are helpful and supportive in nature (Organ, 2018), and less prone to the feelings that are antithetical to OCBs. As a result of these affective links, mindful employees should engage in OCBs more often. Study 2 pursues these predictions, which are complementary to those of Study 1.

### Method

#### Participants and Procedures

As part of an extra credit option that exposed students to different careers, business majors in organizational behavior and leadership classes received extra credit for an assignment that involved recruiting and interviewing managers in the community (not the university) who had been in their jobs for at least 5 years. Approximately 80 students took advantage of this opportunity and they interviewed 5+ people each. After documenting the interviews for extra credit purposes, students emailed a link to a secure, Qualtrics-programmed website with materials for the present study. After completing the study, managers were taken to another, non-linked website where they entered contact information, and this contact information was solely used for verification purposes (i.e., it could not be linked to the relevant data).

Through procedures of this type, we were able to recruit a sample of 329 full-time employees, the vast majority of whom were from the upper Midwest region of the United States. These employees tended to be in their 30s and 40s (*M* age = 40.57) and 43.77% were female. They had been at their current places of employment for an average of 8.06 years and held a variety of positions such as registered nurse, computer tech, certified public accountant, and CEO. Scores from the factual autonomy scale (Spector and Fox, 2003) were consistent with full-time jobs with some managerial responsibilities (*M* = 3.89; *SD* = 0.83; *α* = 0.86).

#### Measures

##### Dispositional Mindfulness

We again assessed mindfulness using the MAAS (*M* = 4.49; *SD* = 0.69; *α* = 0.86), which is thought to capture the core features of mindfulness—namely, greater attentiveness and awareness concerning current experiences (Brown and Ryan, 2003, 2004).

##### Job Stress

Some of the benefits of mindfulness may follow from lesser levels of job stress (Mesmer-Magnus et al., 2017). To examine processes of this type, we administered the well-validated job stress inventory of Cavanaugh et al. (2000). The inventory asks employees how much stress they experience (1 = produces no stress; 5 = produces a great deal of stress) as a function of 16 work-related stressors, some of which are more task-related (e.g., “the number of projects and/or assignments I have”) and some of which concern features of the workplace (e.g., “the lack of job security I have”). A total score averaged across items (*M* = 2.53; *SD* = 0.73; *α* = 0.89).

##### Job Engagement

Mindfulness should reasonably contribute to job engagement, which can be defined in terms of attentiveness and enthusiasm in the conduct of one’s work (Rich et al., 2010). To examine processes of this type, we administered the Saks (2006) engagement scale, which asked employees to rate their agreement (1 = strongly disagree; 7 = strongly agree) with 5 statements indicating job engagement (e.g., “I am highly engaged in this job”) and an average score was computed (*M* = 5.25; *SD* = 1.16; *α* = 0.83).

##### Organizational Citizenship Behaviors

We assessed two forms of OCB using the well-validated scales of Williams and Anderson (1991). One 7 item scale focused on OCBs directed toward individuals (e.g., “help others who have been absent”) and the other 7 item scale focused on OCBs directed toward the organization as a whole (e.g., “conserve and protect organizational property”). Employees indicated whether they performed these behaviors using an agree-disagree rating format (1 = strongly disagree; 7 = strongly agree). In parallel to Study 1, we computed one score to reflect individual-level behaviors (OCB-I: *M* = 5.53; *SD* = 0.83; *α* = 0.78) and another to reflect broader organizational behaviors (OCB-O: *M* = 5.78; *SD* = 0.76; *α* = 0.69).

### Results

#### Correlations Among Variables

Mindful employees were more likely to help coworkers in need (OCB-I), *r* = 0.20, *p* = 0.004, and they were more helpful toward the organization as a whole (OCB-O), *r* = 0.36, *p* < 0.001. The affect-related variables were also consequential. Stress was an inverse predictor of OCB-I frequency, *r* = −0.14, *p* = 0.013, and it was also an inverse predictor of OCB-O frequency, *r* = −0.19, *p* = 0.004. By contrast, employees who were more engaged with their jobs tended to enact both OCB-I, *r* = 0.25, *p* = 0.002, and OCB-O, *r* = 0.24, *p* = 0.003, behaviors with more regularity. Finally, there was some evidence to suggest that the benefits of mindfulness could be affect-linked. Specifically, mindful workers experienced less stress in their jobs, *r* = −0.26, *p* = 0.001, and they were also more engaged with them, *r* = 0.21, *p* = 0.004.

#### Mediational Results Involving Individual-Level OCBs

Mindful employees might help their coworkers more often, in part, *because* they experience less stress and are more engaged with their jobs. To examine this possibility, we performed a series of analyses with the PROCESS-related procedures of Hayes (2013), which uses bootstrapping methods to determine whether mediational pathways are significant. As in Study 1, all of the variables were standardized, which aids magnitude interpretation, and we conducted a comprehensive analysis that considered both job stress and job engagement.

As shown in the top portion of Table 2, job stress and job engagement emerged as plausible mediators of the relationship between mindfulness and OCB-I frequency. Mindfulness was negatively related to job stress (Model 1) and positively related to job engagement (Model 2), the proposed mediators. Further, both of these affect-related mediators were significant when controlling for mindfulness (Model 3) and the predictive power of mindfulness was somewhat reduced when comparing Model 4, in which mindfulness was the sole predictor, to Model 3, in which the proposed mediators were also included. This mediation could be considered partial, though, in that mindfulness was still a significant predictor in the latter model.

**Table 2:** PROCESS model results for organizational citizenship-individual (top panel) and organizational citizenship-organizational (bottom panel), Study 2.

		Outcome	Predictor	*β*	*t*	*p*
*Predictor to Mediator Models*						
	Model 1	J. Stress	Mind.	−0.26	−4.85	<0.01
	Model 2	J. Engage	Mind.	0.21	3.87	<0.01
*Full Mediational Model*						
	Model 3	OCB-I	Mind.	0.12	2.11	0.04
			J. Stress	−0.12	−2.15	0.03
			J. Engage	0.23	4.23	<0.01
*Predictor to Outcome Model*						
	Model 4	OCB-I	Mind.	0.20	3.65	<0.01
*Full Mediation Model*						
	Model 3	OCB-O	Mind.	0.29	5.36	<0.01
			J. Stress	−0.13	−2.43	0.02
			J. Engage	0.19	3.64	<0.01
*Predictor to Outcome Model*						
	Model 4	OCB-O	Mind.	0.36	6.98	<0.01

Mind. = Mindfulness; J. Stress = Job Stress; J. Engage = Job Engagement; OCB-I = Organizational Citizenship-Individual; and OCB-O = Organizational Citizenship-Organizational.

**Figure 2:**
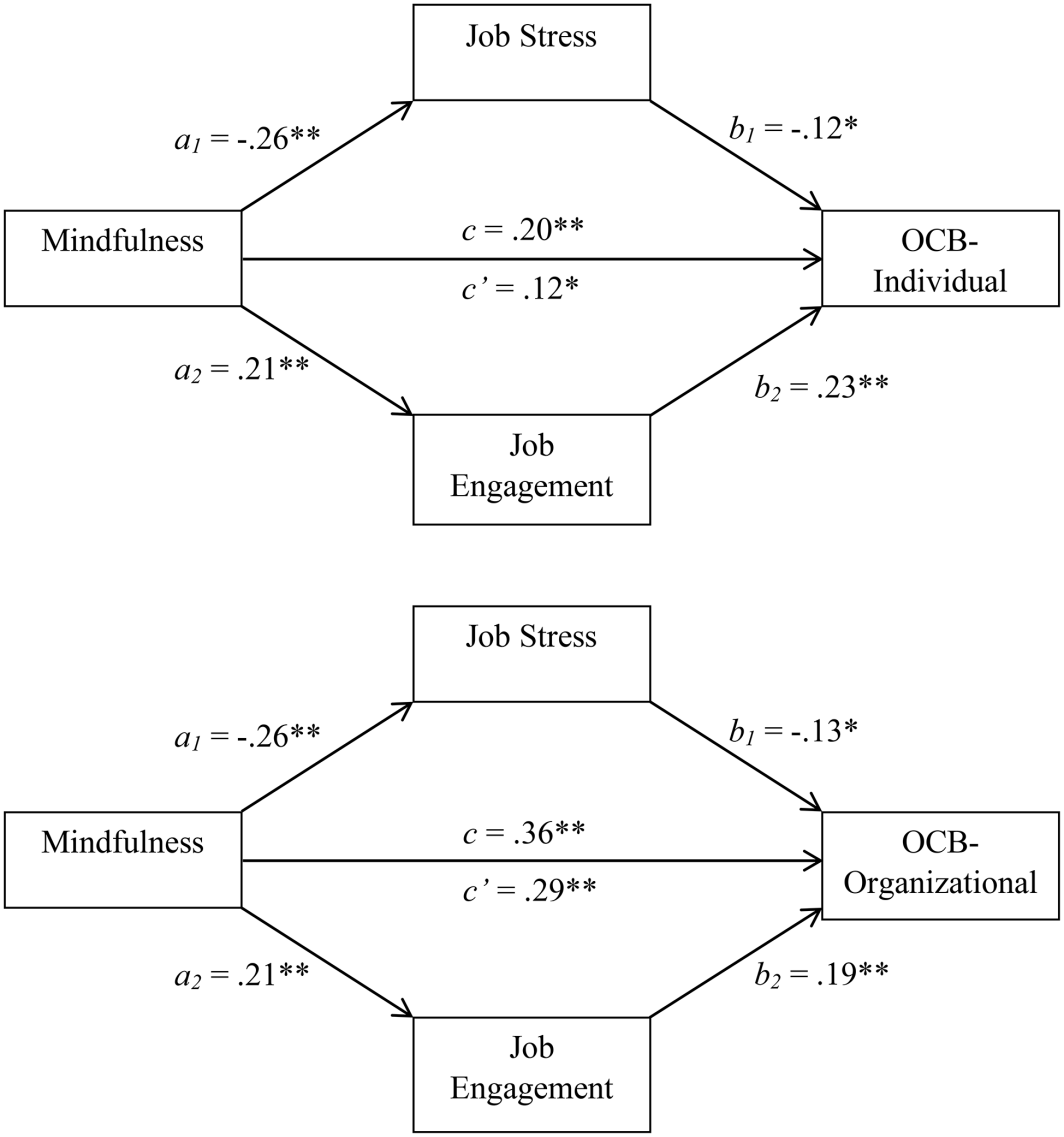
Job stress and job engagement as mediators of the relationship between mindfulness and citizenship behaviors directed toward individuals (**top panel**) and the organization (**bottom panel**), Study 2.

The top panel of Figure 2 displays these results in graphic terms. As shown there, mindful employees tended to experience less stress (path *a_1_*), which was inversely related to individual-level helping rates (path *b_1_*). Mindful individuals were also more engaged with their jobs (path *a_2_*), which tended to support higher helping rates (path *b_2_*). Although mindfulness had a slightly stronger relationship with job stress (path *a_1_*) than with job engagement (path *a_2_*), job engagement (path *b_2_*), relative to job stress (path *b_1_*), tended to be the stronger predictor of OCB-I frequency. Thus, the two mediational pathways appeared somewhat equally strong.

To determine whether the mediational pathways were significant, we turned to the results of the bootstrapping analyses (Hayes, 2013). The mean estimate for the combined *ab* pathways was 0.08 and the 95% BCCI for this estimate was 0.04 to 0.14. Because these figures exclude 0, we can conclude that the mediational model was significant (MacKinnon and Fairchild, 2009). Interestingly, further analyses indicated that both job stress (*a_1_b_1_ M* = 0.03; 95% BCCI = 0.01 to 0.07) and job engagement (*a_2_b_2_ M* = 0.05; 95% BCCI = 0.02 to 0.09) were significant mediators considered in isolation. That is, mindful employees may be more helpful to their colleagues *both* because they are less stressed on the job and because they are more engaged.

The mediation was partial, though. Even when controlling for job stress and job engagement, mindfulness still predicted OCB-I frequency (path *c’* = 0.12). Expressed in other terms, the two mediators together accounted for 39.88% of the relationship between mindfulness and OCB-I frequency (15.52% when considering job stress alone and 24.36% when considering job engagement alone). Independent of affective pathways, then, there may be cognitive pathways as well. For example, mindful employees could be more responsive in part because they are more aware of cases in which their coworkers have need.

#### Mediational Results Involving Organizational-Level OCBs

Similar results were obtained when examining OCB-O frequency. Mindfulness was a positive predictor of OCB-O likelihood (Model 4 in the bottom portion of Table 2). This relationship, however, was somewhat reduced in magnitude when job stress and job engagement were controlled, which themselves remained significant predictors (Model 3 in the bottom portion of Table 2). In parallel to OCB-I frequency, mindfulness was a negative predictor of job stress (path *a_1_* in the bottom portion of Figure 2) and job stress seemed to inhibit OCB-Os (path *b_1_*). Conversely, mindfulness was a positive predictor of job engagement (path *a_2_*) and employees who were more engaged with their jobs performed OCB-Os more frequently (path *b_2_*). Thus, both job stress and/or engagement could mediate the mindfulness/OCB-O relationship.

Consistent with these thoughts, bootstrapping results supported the possibility of mediation. When considering job stress and job engagement together, the estimated *M* for the *ab* pathway was 0.07 and the 95% BCCI excluded 0 (0.03 to 0.12). Thus, the mediational model was significant (MacKinnon and Fairchild, 2009). In addition, significant mediation occurred for both the job stress (*a_1_b_1_ M* = 0.03; 95% BCCI = 0.01 to 0.07) and job engagement (*a_2_b_2_ M* = 0.05; 95% BCCI = 0.02 to 0.08) variables considered alone. Mindfulness also predicted OCB-O occurrence for other reasons, however, as the two mediators together accounted for 20.17% of the relationship between mindfulness and OCB-O frequency (9.19% when considering job stress alone and 10.98% when considering job engagement alone). OCB-Os, like OCB-Is, might therefore benefit from the cognitive, in addition to the affective, features of mindfulness.

### Discussion

Study 2 sought to examine potential relationships between mindfulness and organizational citizenship. In part because job stress tends to undermine citizenship (Greenidge and Coyne, 2014), while job engagement tends to support it (Rich et al., 2010), we hypothesized that mindful employees would engage in organizational citizenship behaviors more often. Study 2 supported this idea and further showed that the mindfulness/OCB relationship was partially mediated by job stress and job engagement. Hence, affect-related variables seem explanatory in accounting for why mindful people are more prosocial (Study 2) as well as less antisocial (Study 1) within the workplace. In this context, though, we must admit that the mediational pathways of Study 2 were not as strong as those in Study 1. This suggests the presence of unmeasured mediators (MacKinnon and Fairchild, 2009) like compassion (Brown and Ryan, 2004) or organizational commitment (Borman and Motowidlo, 1997). Accordingly, the relationship between dispositional mindfulness and OCB merits further attention.

## General Discussion

Brown and Ryan (2003) proposed that the essence of mindfulness is to be attentive and aware of one’s current experiences and that these achievements vary somewhat naturally in the population. Some people habitually think about the past or the future or find it uncomfortable to focus on their raw sensations within the moment (Germer, 2005). Others, by contrast, attune themselves to what is actually happening to them as they go about their daily lives. These individual differences in dispositional mindfulness are reliable and seem to be fairly consequential. Low levels of mindfulness have been implicated in several forms of psychopathology and addiction (Gratz and Tull, 2010), whereas high levels of mindfulness have been linked to better psychological and emotional wellbeing (Brown et al., 2007). Moreover, mindful people seem to be better at emotion regulation (Chambers et al., 2009) and self-regulation (Fetterman et al., 2010).

Mindfulness could be similarly beneficial within the workplace (Reb et al., 2020) and the present studies sought to contribute in this area by focusing on potential relationships between dispositional mindfulness and contextual or voluntary work behavior. It seemed to us that mindful employees would be less prone to deviant workplace behaviors, in part because of the equanimity that is associated with mindfulness (Desbordes et al., 2015), and that mindful employees would also engage in organizational citizenship behaviors more frequently. Two studies supported these ideas. In Study 1, part-time employees who were higher in mindfulness were less prone to deviant workplace behavior and this inverse relationship was mediated by job negative affect. In Study 2, full-time employees who were higher in mindfulness tended to be more helpful toward their organization and coworkers. Further, portions of these relationships could be attributed to two feeling-infused variables—namely, job stress and job engagement.

### Theoretical Implication

Theorists have offered a great diversity of mechanisms to explain how mindfulness could result in behavioral benefits, but a simple possibility is that many of these benefits are likely to follow from the emotional experiences, or feelings, that often guide our behaviors. Mindfulness, through its links to self-efficacy (Charoensukmongkol, 2013), equanimity (Desbordes et al., 2015), and habituation (Germer, 2005), might be expected to decrease the frequency or intensity of negative emotional experiences, which would, in turn, decrease one’s tendencies toward emotional impulsivity (Carver and Johnson, 2018). Conversely, mindful individuals should be more capable of engaging with the environment and savoring pleasant experiences that arise (Kabat-Zinn, 2005) and these feeling-based correlates of mindfulness could contribute to better interpersonal functioning (Pratscher et al., 2018). From the perspective of an *affect mediation model*, that is, many of the behavioral benefits of mindfulness are likely to follow from the feelings that mindfulness produces.

Consistent with this analysis, the employees of the present studies were less prone to job stress or job negative affect and their behaviors appeared to benefit organizational functioning (Podsakoff et al., 2000) for such reasons. In this connection, though, it is worth highlighting one difference between Studies 1 and 2. The Study 2 job stressor measure asked people how much stress they experience in response to specific features of the job (e.g., a lack of job security). Because some of these features will reflect the nature of the job rather than one’s subjective reaction to it, it is probable that the results of Study 2 somewhat underestimate the extent to which mindfulness is protective against stress, conceptualized as a subjective experience. Consistent with this possibility, the relationship between mindfulness and JNA, in Study 1, was stronger than the similar relationship that was observed in Study 2. Further studies might explore this distinction between event-based and subjective measures of stress to see whether mindfulness matters more in the latter case than in the former. This should reasonably be the case (Kabat-Zinn, 2005) and our affect mediation model should also result in stronger pathways when more subjective measures are used.

The results of the two studies also revealed differences among measures of positive affect and motivation. Although mindfulness did not predict JPA in Study 1, mindfulness did predict job engagement in Study 2. There are several potential differences between these measures. The markers of the JPA measure focus on high arousal positive states and mindfulness might tend to promote calmer types of positive affect (Rau and Williams, 2016). Perhaps more centrally, the JPA measure focuses on episodic or time-limited experiences of excitement, pride, etc. By contrast, the job engagement measure focuses on the more protracted sorts of feelings that can follow from the consistent application of oneself to one’s job (Saks, 2006). Mindfulness may be particularly conductive to these sorts of task-oriented feelings of positive affect and engagement, which are amplified by the sustained type of attention that mindfulness provides (Robinson and Tamir, 2011). Given that job engagement predicts organizational commitment and citizenship (Saks, 2006), these sorts of feelings are likely to contribute to job performance in some larger sense, including its contextual aspects (Borman and Motowidlo, 1997).

Consistent with these ideas, Study 2 found that mindful employees were more engaged and engagement, in turn, predicted organizational citizenship (e.g., protecting organizational resources). That is, the affective profile of mindfulness was one that favored organizational citizenship and the mindfulness/OCB relationship could be partly understood in these affect-related (and affect-mediated) terms. Similarly, Study 1 found that mindful employees were less prone to negative affect on the job (JNA) and lower levels of JNA were linked to lower levels of workplace deviance. Hence, mindful employees seem to be better employees, in a contextual sense (Borman and Motowidlo, 1997), because mindfulness is linked to the sorts of affective experiences that often motivate the relevant behaviors (Greenidge and Coyne, 2014). These results make a further case for the utility of mindfulness at work (Reb et al., 2020) and they also elucidate a class of relevant mechanisms. In this connection, the results offer new evidence for the affect mediation perspective of mindfulness-related benefits.

In addition, and although mindfulness is often conceptualized in terms of intrapersonal (within-person) mechanisms (Brown and Ryan, 2004), the present results contribute to the idea that mindfulness has predictable interpersonal consequences. Mindful employees were less hostile in the workplace (Study 1) and they were more helpful toward their fellow employees (Study 2). These results join a handful of other recent findings (e.g., Pratscher et al., 2018; Karremans et al., 2020) in pointing to an interpersonal reconceptualization of what mindfulness does to one’s behaviors. This reconceptualization accords with the teachings of the Dalai Lama (Dalai Lama and Ekman, 2008).

### Practical Implications

Acts of deviance or organizational citizenship tend not to comprise the technical core of one’s job, but employers care about such behaviors nonetheless (Werner, 2000). Indeed, supervisors tend to consult task performance and contextual performance somewhat equally when evaluating their employees (Borman, 2004), presumably because helpful, non-hostile employees contribute to organizational functioning in ways that go beyond task production narrowly considered (Podsakoff et al., 2000). Personality tests are useful predictors of the sorts of behaviors that the person is likely to exhibit in the workplace (Nikolaou and Foti, 2018) and dispositional mindfulness may have particular value in this context. We say this in part because the Mindful Attention and Awareness Scale that was used in the present studies does not ask individuals to report on socially sensitive features of their personalities. Rather, it probes for relatively mundane losses of attention and awareness, whether due to distraction or difficulties in sustaining attention (Brown and Ryan, 2003). As shown in the present studies, these tendencies matter quite a bit, but their mundane nature may bypass the sorts of defensive processes that can occur when socially sensitive topics are reported on. If so, instruments capturing dispositional mindfulness might have particular value in recruitment and selection contexts.

In further extending the analysis, the present results suggest that mindfulness may be a particularly useful set of capacities within jobs in which deviant behaviors would be particularly problematic (Bennett and Robinson, 2003) and/or in which citizenship behaviors are closely bound to the tasks that individuals are being asked to perform (Podsakoff et al., 2000). Included within the latter category, for example, would be jobs that involve customer service (Dane and Brummel, 2014), team coordination (Jordan et al., 2007), or managerial responsibilities (Dixon, 2015). Employee variations in mindfulness could be used as a basis for assigning individuals to roles or tasks within the organization. The results further suggest, though, that it would make sense for organizations to attend to the mediators that were highlighted in the present research. Reducing levels of negative affect on the job could protect the organization against deviant behaviors (also see Bennett and Robinson, 2003) and inculcating higher levels of employee engagement is likely to produce multiple benefits to the organization as a whole (Rich et al., 2010). In other words, there are multiple ways in which the present findings could be used to craft better-functioning workplaces.

Mindfulness, finally, is not fully dispositional in that it can be systematically trained through contemplative practices (Creswell, 2017). Such interventions are effective and this appears to be true concerning workplace-based interventions as well as those practiced outside of the workplace. For example, a meta-analysis from Bartlett et al. (2019) showed that mindfulness-based interventions for the workplace were effective in reducing employee stress levels (*g* = 0.56) and in increasing employee wellbeing levels (*g* = 0.46). Because such experiences matter for workplace productivity (Judge et al., 2008) as well as contextual behavior (Organ, 2018), the present results, in combination with recent intervention work (Bartlett et al., 2019), point to the organizational benefits that are likely to follow from such efforts (also see Charoensukmongkol, 2013; Hyland et al., 2015).

### Limitations and Future Directions

Our affect-mediation framework has implications for the types of workplace behaviors that mindfulness should relate to. Mindfulness should predict classes of behavior that are motivated by negative affect (e.g., withdrawal) and it should predict other classes of behavior that stem from involvement in the job (e.g., helping coworkers in need). Mindfulness might be less consequential in predicting behaviors that lack an emotional basis to them, perhaps including instrumental aggression or manipulation. On this point, however, many scholars believe that mindfulness facilitates compassion (e.g., Dalai Lama and Ekman, 2008) and compassion would be broadly inconsistent with manipulative or hostile behaviors within the workplace.

Turning to a different point, we assessed what is arguably the core component of mindfulness—namely, attention and awareness of current experience (Brown and Ryan, 2004). Nonetheless, there are also multi-dimensional scales for mindfulness (e.g., Baer et al., 2004) and many conceptions of mindfulness include both attention-awareness and acceptance (Bishop et al., 2004). Attention-awareness and acceptance are likely to function similarly in that both should be linked to lower levels of negative emotionality (Lindsay et al., 2018). However, there may be subtle differences that merit future attention. For example, the acceptance component of mindfulness could have a stronger relationship with job stress, and a weaker relationship with job engagement, than was observed for attention-awareness in Study 2. Dissociations of this type would be of interest (Cameron and Fredrickson, 2015; Hyland et al., 2015).

Our focus was also on extra-role behaviors rather than in-task performance. Thus, future research is necessary in examining whether mindfulness facilitates task performance (for a review, see Sutcliffe et al., 2016; for an interesting qualitative analysis, see Lyddy and Good, 2017). Perhaps of more significance, it might be useful to replicate the present results using other sorts of assessment techniques. Along these lines, we asked participants in Study 1 to report on their own deviant behaviors, following considerable precedent (Spector and Fox, 2002), but it should be possible to supplement such reports with coworker perceptions (Fox et al., 2007). Similarly, supervisor or coworker perceptions of organizational citizenship could have merit. Regardless, it should be emphasized that self-reports concerning these behaviors seem to have the same correlates as observer reports (Berry et al., 2012) while displaying greater sensitivity (Berry et al., 2012; Carpenter et al., 2014).

In Study 1, the sample involved part-time student employees. Student employees constitute a primary component of many workplaces (Moorman and Harland, 2002) and previous investigations suggest that many organizational processes operate similarly across the part-time/full-time continuum (Highhouse and Gillespie, 2009). Nonetheless, we do suggest that it would be useful to replicate the results of Study 1 within a sample of full-time employees. This need does not extend to Study 2, which surveyed employees with full-time jobs.

There were reasons why we employed the designs that we did, but such decisions merit further discussion. As assessed, mindfulness is a stable personality trait that is likely to precede and give rise to the affective and behavioral manifestations that were focused on (Goodman et al., 2015). Furthermore, it was important to assess affect and behavior contemporaneously as the theoretical perspectives that we borrowed from (e.g., Spector, 2011) emphasize the manner in which current feeling states influence current workplace behaviors (Weiss and Cropanzano, 1996). Accordingly, and despite the fact that we used cross-sectional assessment techniques, the mediational models ordered the variables in a theoretically sensible way (MacKinnon and Fairchild, 2009). Regardless, lagged or longitudinal designs could also be used in future research, particularly in examining more state-like links among mindfulness, affect, and behavior, which are likely to be present as well (Hafenbrack et al., 2020).

Given our designs, though, unmeasured third variables could have played some role in the findings. Although the trait of mindfulness appears to be a unique one, it does overlap with other components of personality like neuroticism and conscientiousness (Rau and Williams, 2016). Also, there is the possibility that certain features of the workplace—such as how stressful and demanding objective conditions are—could have contributed to some of the relationships that were observed. Nonetheless, it is our contention that the findings in total implicate processes that are central to the manner in which dispositional mindfulness is likely to operate.

### Conclusion

Employees who were higher in trait mindfulness were less likely to engage in deviant workplace behaviors (Study 1) and they performed organizational citizenship behaviors more frequently (Study 2). Further key results established that these relationships had an affective basis to them. For example, mindful employees were less likely to feel stress, and they reported higher levels of engagement with their jobs, and these feelings partially explained their greater prosocial tendencies (Study 2). Thus, the findings indicate that dispositional components of mindfulness benefit workplace functioning and that some of these benefits follow from affective states that are less aversive and more engaged.

## Data Availability Statement

The raw data supporting the conclusions of this article will be made available by the authors, without undue reservation.

## Ethics Statement

The studies involving human participants were reviewed and approved by North Dakota State University Institutional Review Board. The patients/participants provided their written informed consent to participate in this study.

## Author Contributions

MR and SK jointly conducted the research. MR played a primary role in writing the paper. SK played a primary role in data analysis. All authors contributed to the article and approved the submitted version

## Conflict of Interest

The authors declare that the research was conducted in the absence of any commercial or financial relationships that could be construed as a potential conflict of interest.

## Publisher’s Note

All claims expressed in this article are solely those of the authors and do not necessarily represent those of their affiliated organizations, or those of the publisher, the editors and the reviewers. Any product that may be evaluated in this article, or claim that may be made by its manufacturer, is not guaranteed or endorsed by the publisher.
